# IgG4-Related Disease: Current and Future Insights into Pathological Diagnosis

**DOI:** 10.3390/ijms26115325

**Published:** 2025-06-01

**Authors:** Marlon Arias-Intriago, Tamar Gomolin, Flor Jaramillo, Adriana C. Cruz-Enríquez, Angie L. Lara-Arteaga, Andrea Tello-De-la-Torre, Esteban Ortiz-Prado, Juan S. Izquierdo-Condoy

**Affiliations:** 1 One Health Research Group, Universidad de las Américas, Quito 170124, Ecuador; 2 Department of Pathology, Icahn School of Medicine at Mount Sinai West, New York, NY 10019, USA; 3 Departamento de Hematología, Hospital de la Policía, Quito 170517, Ecuador; 4 Facultad de Ciencias de la Salud, Universidad del Quindío, Armenia 630004, Colombia; 5 Facultad de Ciencias de la Salud, Universidad del Cauca, Popayán 190003, Colombia

**Keywords:** IgG4-related disease, diagnostic criteria, histopathology, biomarkers, differential diagnosis

## Abstract

Immunoglobulin G4-related disease (IgG4-RD) is a systemic fibroinflammatory condition marked by tumefactive lesions, IgG4+ plasma cell-rich infiltrates, storiform fibrosis, and obliterative phlebitis. Its multisystem involvement and overlap with malignancies, infections, and immune disorders complicate diagnosis despite recent classification advances. This study summarizes diagnostic challenges, highlights the role of histopathology as per the 2019 classification criteria established by the American College of Rheumatology and the European League Against Rheumatism (ACR/EULAR), and explores emerging tools to improve diagnostic accuracy. ACR/EULAR classification emphasizes three cardinal histopathological features (storiform fibrosis, obliterative phlebitis, or dense lymphoplasmacytic infiltrates) combined with an IgG4+/IgG+ plasma cell ratio >40% and organ-specific IgG4+ thresholds. While serum IgG4 levels are often elevated, their poor specificity necessitates confirmatory biopsy. Diagnostic limitations include sampling variability due to patchy fibrosis, interobserver discrepancies in immunohistochemical interpretation, and differentiation from mimics like lymphoma. Emerging solutions incorporate novel biomarkers (plasmablasts, anti-annexin A11) and advanced techniques (flow cytometry, digital pathology). Future research directions should focus on AI-assisted pattern recognition, multi-omics profiling, and organ-specific criteria refinement. While histopathology remains the diagnostic cornerstone, a multidisciplinary approach integrating clinical, radiological, and laboratory data is vital. Innovations in biomarkers promise improved diagnostic accuracy and personalized care, balancing novel advancements with foundational pathological evaluation.

## 1. Introduction

Immunoglobulin G4-related disease (IgG4-RD) is a chronic, immune-mediated fibroinflammatory condition characterized by tumefactive lesions, dense lymphoplasmacytic infiltrates rich in IgG4-positive plasma cells, storiform fibrosis, and obliterative phlebitis [1]. This multisystemic disease can affect virtually any organ, with predilection for the pancreas, kidneys, orbital adnexal structures, salivary glands, and retroperitoneum [2,3]. Due to its broad clinical manifestations, nonspecific serological markers, and histopathological overlap with malignancies, infections, and other autoimmune diseases, diagnosis of IgG4-RD often poses significant challenges [1].

Over the past two decades, the understanding of IgG4-RD has evolved considerably. Initially recognized through disparate organ-specific conditions such as autoimmune pancreatitis (AIP), Mikulicz disease, and Riedel thyroiditis, these disorders were later unified under a single diagnostic framework based on shared histopathological features—namely, IgG4-positive plasma cell infiltration and storiform fibrosis [4]. This conceptual unification marked a pivotal step toward appreciating the systemic nature of IgG4-RD and standardizing diagnostic and therapeutic approaches [4].

Although the global epidemiology of IgG4-RD remains incompletely defined due to underrecognition and diagnostic variability, available data indicate predominance among males (approximately 2:1) and a median age at diagnosis between 60 and 70 years [5]. In Japan, the prevalence of AIP, a prototypical manifestation of IgG4-RD, rose from 2.2 to 4.6 cases per 100,000 between 2007 and 2011, probably reflecting heightened clinical awareness and improved diagnostic capacity [5]. Pediatric cases are rare, and familial clustering is exceptionally uncommon [6].

Despite growing recognition, IgG4-RD is still frequently misdiagnosed, especially in cases presenting with mass-like lesions in the absence of systemic symptoms. While IgG4 itself is not considered directly pathogenic, recent studies have implicated cytotoxic CD4^+^ SLAMF7^+^ T cells as key drivers of tissue injury and fibrosis via profibrotic cytokines and cytolytic molecules [7]. Although candidate autoantigens—such as annexin A11 and galectin-3—have been proposed, definitive antigenic triggers remain elusive [8,9].

Histopathological remains central to diagnosis, with biopsy considered the gold standard. The 2019 classification criteria established by the American College of Rheumatology and the European League Against Rheumatism (ACR/EULAR) formalized key diagnostic elements, integrating clinical, serological, and histopathological data to improve diagnostic precision [10].

This study aims to review current and emerging insights relevant to the pathological diagnosis of IgG4-RD. By synthesizing recent advances in histopathology, immunohistochemistry, and molecular diagnostics, it seeks to support pathologists in enhancing diagnostic accuracy and recognizing the evolving spectrum of IgG4-RD.

## 2. Diagnostic Challenges

Despite the presence of well-established pathological hallmarks, diagnosis of IgG4-RD remains complex due to several interrelated factors. The disease exhibits a highly variable clinical presentation, largely determined by the specific organs involved [2]. This heterogeneity can hinder early clinical suspicion, particularly when organ involvement is limited or atypical. Moreover, IgG4-RD often follows an indolent, relapsing course and may remain asymptomatic for extended periods, contributing to delays in diagnosis and increasing the risk of progressive fibrosis and irreversible organ dysfunction [2]. Patients frequently present with non-specific signs and symptoms that overlap with a broad range of other conditions, further complicating the diagnostic process [11]. Given its potential to affect virtually any anatomical site, clinicians across diverse medical specialties must maintain a high index of suspicion to recognize the disease promptly [1].

Diagnostic complexity increases when IgG4-RD presents as a single-organ disorder [1]. In such scenarios, the lack of systemic features often leads to misclassification as malignancy, infection, or localized inflammatory disease. Although elevated serum IgG4 levels are found in a significant proportion of patients, their diagnostic utility is limited. According to Baker et al., the PPV of serum IgG4 concentrations exceeding five times the ULN for diagnosing IgG4-RD was 75.4% (95% CI: 68.7–81.3), which supports the emphasis placed on markedly elevated serum levels in the ACR/EULAR classification criteria. However, approximately 25% of individuals with such elevations were ultimately diagnosed with other conditions, highlighting the importance of a broad differential diagnosis [12]. Furthermore, a notable proportion of patients with biopsy-proven IgG4-RD exhibit normal serum IgG4 levels [1], and serum concentrations do not reliably correlate with disease severity or the extent of organ involvement. Elevated serum IgG4 is also non-specific and may occur in various other contexts, including autoimmune diseases, lymphomas, and chronic infections, thereby limiting its specificity [4]. As such, reliance on serum IgG4 levels alone is insufficient and may result in diagnostic misinterpretation.

Serum IgG4 levels can also be influenced by physiological and pathological conditions unrelated to IgG4-RD. For example, patients undergoing allergen-specific immunotherapy frequently experience 10–100-fold increases in IgG4 concentrations, which are temporally associated with the induction of immune tolerance. These elevations are accompanied by IL-10-mediated suppression of total and allergen-specific IgE, a reduced IgE/IgG4 ratio, and a shift toward tolerogenic Treg responses. IL-10-secreting Tregs promote B-cell class switching to IgG4 via mechanisms involving GITR engagement and TGF-β. In this context, IgG4 serves as a “blocking” antibody that inhibits IgE-mediated histamine release and prevents immediate hypersensitivity reactions. Conversely, in the tumor microenvironment, chronic antigen exposure under Th2 (IL-4/IL-13) and IL-10 signaling drives local IgG4 production, which correlates with poor prognosis in malignancies such as melanoma and cholangiocarcinoma. Tumor-associated IgG4 exhibits low complement activation, preferential binding to the inhibitory FcγRIIb receptor, and reduced capacity for ADCC/ADCP, effectively antagonizing IgG1-mediated antitumor responses. Additionally, IgG4-polarized macrophages acquire an M2b-like phenotype with high CCL1 and IL-10 production, which facilitates recruitment of CCR8+ Tregs and sustains an immunosuppressive tumor milieu [13]. These observations highlight the dualistic nature of IgG4-mediated immunomodulation, which may be beneficial in AIT but detrimental in oncologic contexts.

Histopathological and immunohistochemical findings, while central to the diagnostic process, are also not exclusive to IgG4-RD. The hallmark lymphoplasmacytic infiltrate enriched with IgG4-positive plasma cells can be observed in a range of other pathological conditions. Obtaining representative tissue samples with characteristic features remains a significant challenge. Minimally invasive procedures, such as endoscopic or needle biopsies, may yield insufficient or non-representative material [14]. Additionally, the patchy distribution of storiform fibrosis can lead to sampling error, with biopsies missing key diagnostic areas [11]. Lymph node biopsies, despite frequent involvement, may not consistently display the storiform fibrosis or obliterative phlebitis seen in other organs [4], reducing their diagnostic reliability.

A further challenge lies in the disease’s capacity to mimic a wide spectrum of both benign and malignant conditions, clinically and radiologically. IgG4-RD shares overlapping features with numerous autoimmune, infectious, and neoplastic diseases, necessitating thorough differential diagnosis [4]. In certain clinical contexts, the coexistence or sequential development of IgG4-RD with non-IgG4-related conditions can obscure the diagnostic landscape and confound clinical interpretation [15]. Collectively, these diagnostic pitfalls often lead to delayed recognition, which can adversely affect patient outcomes by allowing disease progression before appropriate treatment is initiated [2].

## 3. Pathological Diagnosis

The future of IgG4-RD diagnosis will depend on a multidisciplinary precision medical approach that combines histopathology, immunoprofiling, and advanced imaging to achieve timely and individualized patient care [15]. Due to the significant overlap between IgG4-RD and mimicking conditions, the development of standardized diagnostic criteria was essential. This effort led to the 2019 ACR/EULAR classification criteria for IgG4-RD, which were groundbreaking as the first for any rheumatic disease to incorporate absolute exclusion criteria. These criteria integrate clinical, serological, imaging, and pathological features to improve diagnostic sensitivity and specificity [10].

Pathologists play a crucial role in accurate diagnosis, as highlighted by the ACR/EULAR consensus, which underscores that biopsy remains necessary in many cases to confirm IgG4-RD and exclude mimics [2,16]. Studies supporting these criteria demonstrated a substantial drop in diagnostic sensitivity when pathology data or serum IgG4 levels were omitted. While the criteria allow diagnosis without biopsy in straightforward cases—based on clinical, serologic, and radiologic findings—histopathology remains indispensable in complex or ambiguous presentations [11].

The 2019 ACR/EULAR criteria employ a weighted scoring system based on the presence or absence of specific clinical and laboratory features. The role of the pathologist in applying these criteria is summarized in Table 1. Pathologists not only ensure diagnostic accuracy through meticulous evaluation but also collaborate with clinicians and radiologists to integrate findings into a cohesive diagnostic framework [10,16].

During the development and validation of the ACR/EULAR criteria, the expert consensus panel prioritized specificity to create homogenous research cohorts, although this approach came at the expense of sensitivity. In two independent validation cohorts, the criteria demonstrated excellent specificity at 99.2% and 97.8%, with corresponding sensitivities of 85.5% and 82.0% [17]. However, subsequent phenotype-based analysis by Wallace et al. identified four major clinical subgroups: pancreato–hepato–biliary; retroperitoneum; aorta, head, and neck-limited; and Mikulicz with systemic involvement. Notably, the more localized phenotypes—retroperitoneum (27.8%) and aorta, head, and neck-limited (60.9%)—showed substantially lower fulfillment rates of the criteria [18]. This suggests that reduced weighting for certain organ involvements and lower serum IgG4 concentrations in these subgroups may diminish sensitivity and introduce a classification bias favoring more systemic, florid disease presentations [17].

Further evaluation by Kogami et al. found that the ACR/EULAR criteria achieved a sensitivity of 88.1% and a specificity of 87.5% for diagnosing IgG4-RD in a Japanese cohort. In contrast, the 2020 revised comprehensive diagnostic (RCD) criteria demonstrated a higher sensitivity of 100% but a lower specificity of only 50% within the same population. This contrast underscores the differing priorities of the two frameworks: while the ACR/EULAR criteria are optimized for specificity, ensuring rigorous exclusion of disease mimics and supporting research cohort uniformity, the RCD criteria are tailored to maximize sensitivity, enabling broader identification of diverse clinical phenotypes and facilitating diagnosis in early or limited-stage disease [19].

### 3.1. Pathological Hallmarks of IgG4-RD

Several key microscopic features have been consistently identified across affected organs. One of the most characteristic findings is a dense lymphoplasmacytic infiltrate, consisting of numerous lymphocytes and polyclonal plasma cells within the tissue [1]. This infiltrate reflects the chronic inflammatory nature of the disease and is typically prominent in all involved sites (Table 2).

Accompanying this inflammatory infiltrate is storiform fibrosis, a highly suggestive pattern of collagen deposition. In this pattern, fibrotic tissue is arranged in a swirling or whorled configuration that radiates around the inflammatory foci, resembling the spokes of a cartwheel [1]. This unique fibrotic architecture is widely regarded as a hallmark of the disease and contributes significantly to its histological recognition (Table 2).

Another major diagnostic feature is obliterative phlebitis, which refers to the inflammation-induced narrowing or occlusion of small- to medium-sized veins. This vascular involvement is considered particularly specific to IgG4-RD and can be seen in many, though not all, affected organs [1] (Table 2).

The identification of IgG4-positive plasma cells through immunohistochemistry is an essential component of the diagnostic process. An increased absolute number of these cells, along with a high IgG4-to-total IgG ratio, strengthens the likelihood of IgG4-RD. Furthermore, an IgG4/IgG ratio greater than 40% is often considered a strong indicator of disease (Table 2) [20].

Other histological features may support the diagnosis when present. Mild to moderate eosinophilia is frequently observed within the infiltrate and, although not specific, may be a helpful ancillary finding [21]. Conversely, the absence of tissue necrosis, neutrophil-rich inflammation, or granuloma formation serves to distinguish IgG4-RD from other chronic inflammatory or infectious diseases [21]. A recognized challenge in histological diagnosis is the patchy distribution of storiform fibrosis, which may lead to sampling errors, particularly when biopsies are small or taken from sites with variable involvement [11].

Consensus guidelines emphasize that a definitive histopathological diagnosis typically requires the presence of at least two of the three principal features—lymphoplasmacytic infiltrate, storiform fibrosis, and obliterative phlebitis—together with a demonstrable increase in IgG4-positive plasma cells and a supportive IgG4/IgG ratio [4]. Given the diagnostic complexity and potential for overlap with other conditions, biopsy confirmation remains strongly recommended before initiating treatment [22].

### 3.2. Immunohistochemistry

Immunohistochemistry plays a vital role in confirming the diagnosis of IgG4-RD by visualizing and quantifying IgG4-positive plasma cells within the tissue [4]. IHC also aids in differentiating IgG4-RD from mimics [23], particularly in organ systems such as the thyroid and biliary tract, where clinical and radiologic features may be non-specific [14].

A key diagnostic criterion is the increased number of IgG4-positive plasma cells, frequently accompanied by an elevated IgG4+/IgG+ plasma cell ratio [1]. According to consensus guidelines, thresholds for diagnosis vary by organ; for example, ≥10 IgG4+ plasma cells per high-power field (HPF) is suggested for the pancreas, whereas >50 IgG4+ cells/HPF may be required in other tissues. Additionally, a ratio of IgG4+/IgG+ plasma cells exceeding 40% is generally considered strongly supportive of IgG4-RD [1]. In certain cases, in situ hybridization can be employed as a complementary method to evaluate IgG4/IgG ratios [13], particularly when IHC is inconclusive or compromised, with a threshold >30% potentially significant (Table 2) [10].

Despite its diagnostic utility, IHC has important limitations. The presence of IgG4-positive plasma cells is not pathognomonic and may be observed in a range of inflammatory, infectious, and neoplastic conditions [3]. Therefore, the interpretation of IHC must be contextualized within the broader clinical and histopathological picture, including architectural features such as storiform fibrosis and obliterative phlebitis [15]. In atypical presentations where classic histologic features are absent, reliance on elevated IgG4+ counts alone may lead to misclassification [10,16].

Technical challenges also impact the reliability of IHC. Variability in staining quality, tissue heterogeneity, and sample size—particularly in small or superficial biopsies—can hinder accurate quantification [24]. Moreover, the standard threshold of ≥10 IgG4+ cells/HPF may be difficult to apply consistently due to interobserver variability in field selection and counting methodology. In practice, pathologists often use semi-quantitative descriptors (e.g., “at least 10/HPF”) to accommodate these uncertainties [16].

Given these limitations, a comprehensive and integrated diagnostic approach is essential. While IHC provides valuable quantitative data, it must be interpreted alongside histological architecture, clinical findings, and, when necessary, ancillary techniques such as ISH. Accurate diagnosis ultimately relies on the expertise of experienced pathologists and multidisciplinary correlation to ensure diagnostic precision and to avoid either over- or under-diagnosis of IgG4-RD [3,11,15].

### 3.3. Future Directions

A deeper understanding of disease pathogenesis, including the identification of key autoantigens and the functional role of IgG4, is critical to the development of more targeted diagnostic assays [4]. Current research focuses on identifying molecular biomarkers beyond serum IgG4, such as circulating plasmablasts, specific autoantibodies, and other serologic or cellular markers [25].

Follicular helper CD4+ T (Tfh) cells play a critical role in IgG4 production by B cells in IgG4-RD. Recent studies showed that SLAMF7+CD4+ T cells are an important pathological driver of IgG4-RD. SLAMF7 is a member of the SLAM (signaling lymphocyte activation molecule) family of receptors expressed on immune cells with cytotoxic properties, such as NK cells and CD8+ T cells. Intriguingly, depletion of B cells leads to decrease in the number of SLAMF7+ CD4+ cytotoxic T cells in IgG4-RD, suggesting that the generation of these cytotoxic T cells is required to assist the B cells or plasmablasts. It is thus likely that in addition to IgG4 secretion, B cells exert Ab-independent functions such as antigen presentation towards T cells [26].

**Figure 1:**
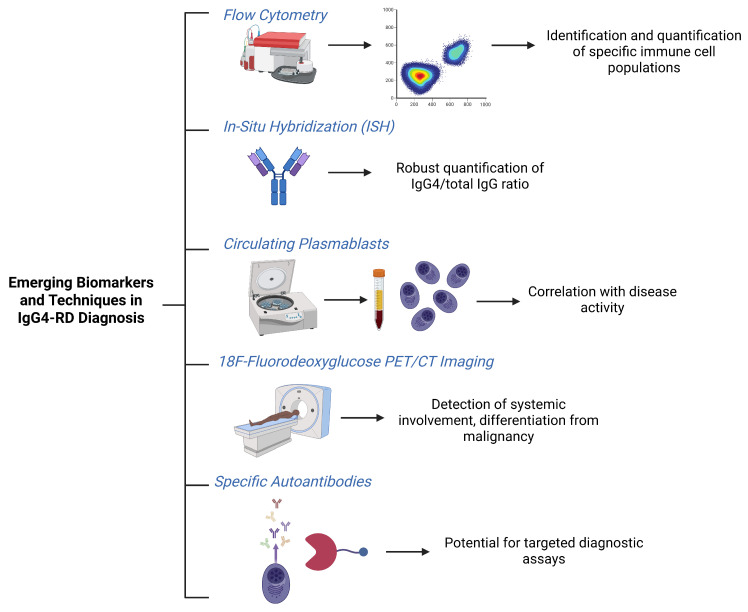
Emerging biomarkers and techniques in IgG4-RD diagnosis.

Innovative diagnostic methodologies are also contributing to improved tissue-based evaluation. Flow cytometry has emerged as a valuable technique for identifying and quantifying pathogenic immune cell subsets, offering potential applications in both diagnosis and disease monitoring [12]. In situ hybridization has demonstrated strong concordance with immunohistochemistry for quantifying IgG4/total IgG ratios and may serve as a robust adjunct in cases where IHC results are ambiguous or affected by limitations in the sampling (Figure 1) [20].

Furthermore, the integration of multi-omics approaches encompassing genomics, transcriptomics, and proteomics holds promise for unraveling the molecular architecture of IgG4-RD. These technologies could facilitate the identification of diagnostic biosignatures, enable disease subclassification, and provide insights into disease susceptibility and progression [5].

Looking ahead, the development of non-invasive diagnostic tools represents a key frontier. Advances in high-resolution imaging modalities with enhanced specificity, along with next-generation serological assays, may reduce dependency on invasive biopsies, particularly in anatomically challenging or high-risk cases [11]. Collectively, these innovations are expected to reshape the diagnostic paradigm of IgG4-RD, enabling earlier detection, improved accuracy, and more individualized patient care.

Genetic and immunological research is also advancing our understanding of the pathophysiology of IgG4-RD, with ongoing studies aimed at identifying potential genetic markers that could improve diagnosis or risk stratification [3]. Beyond serum IgG4 concentrations, which have limited specificity, circulating plasmablasts have gained attention as biomarkers due to their correlation with disease activity [25]. The discovery of autoantibodies against annexin A11 and galectin-3 holds promise for the development of more targeted serological assays in the future [5].

Advanced imaging techniques are also playing an increasingly pivotal role. Combined 18F-FDG PET/CT is particularly valuable in assessing disease extent, identifying occult organ involvement, and guiding biopsy procedures. Moreover, its capacity to differentiate IgG4-RD from malignancy based on characteristic uptake patterns adds to its diagnostic utility [23,27]. Conventional imaging tools such as CT and MRI remain essential for evaluating organ involvement and lesion morphology [11]. In pancreatobiliary presentations, endoscopic ultrasound (EUS) and intraductal ultrasound (IDUS) enhance visualization of ductal structures and facilitate tissue sampling [14].

## 4. Differential Diagnosis

Accurate diagnosis of IgG4-RD depends on the ability to distinguish it from a wide range of conditions that can present with overlapping clinical, radiological, or histopathological features [25]. This process requires a thorough, multidisciplinary evaluation that integrates clinical presentation, serological data, imaging findings, and, most importantly, histopathological and immunohistochemical evidence [28,29].

Involvement of the pancreas and biliary tract is among the most frequently misinterpreted presentations of IgG4-RD. IgG4-related sclerosing cholangitis (IgG4-SC) may closely resemble primary sclerosing cholangitis (PSC) and cholangiocarcinoma. Both IgG4-SC and PSC can cause bile duct strictures; however, histological identification of a dense lymphoplasmacytic infiltrate with abundant IgG4-positive plasma cells and, when present, elevated serum IgG4 levels supports a diagnosis of IgG4-SC. Radiological features such as uniform wall thickening and enhancement of the bile ducts on CT imaging may further aid distinction [14]. Similarly, AIP, a hallmark manifestation of IgG4-RD, often mimics pancreatic adenocarcinoma. In such cases, correlation between imaging, serologic findings, and especially, histopathology is essential to avoid misdiagnosis and unnecessary surgical intervention [11].

In the head and neck region, IgG4-related dacryoadenitis and sialadenitis must be differentiated from Sjögren’s syndrome, sarcoidosis, and benign obstructive causes such as sialolithiasis [21]. While all may present with glandular swelling and sicca symptoms, histopathological examination remains critical. IgG4-RD typically exhibits a more intense IgG4-positive plasma cell infiltration and higher IgG4/IgG ratio compared with Sjögren’s syndrome, and it lacks the lymphoepithelial lesions typical of the latter [30]. Sarcoidosis can be distinguished by the presence of non-caseating granulomas, whereas sialolithiasis generally lacks any significant inflammatory or fibrotic histological features [3].

**Figure 2:**
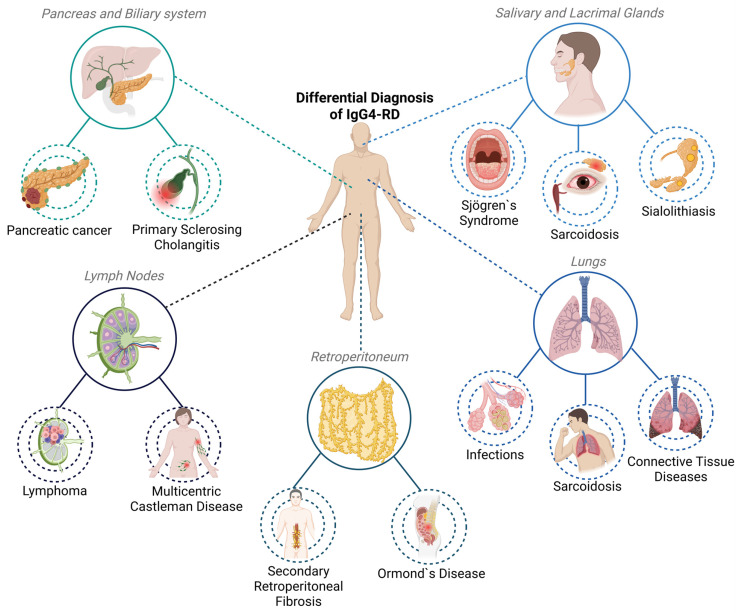
Organ-specific differential diagnoses of IgG4-RD.

Lymphadenopathy is a common manifestation of IgG4-RD and frequently mimics lymphoproliferative disorders such as Hodgkin and non-Hodgkin lymphoma, multicentric Castleman disease, and reactive hyperplasia. Immunohistochemical analysis for IgG4 is useful in these contexts, although it is important to recognize that lymph node involvement in IgG4-RD may not always exhibit the characteristic storiform fibrosis or obliterative phlebitis seen in other organs [4]. Therefore, interpretation should always consider the broader clinical and serological context (Figure 2).

Retroperitoneal fibrosis associated with IgG4-RD may resemble idiopathic, secondary, or malignant causes of retroperitoneal fibrosis. While imaging is valuable for anatomical delineation, histopathology remains the definitive method for distinguishing IgG4-RD from other etiologies [11]. In the lungs, IgG4-RD can present radiological features similar to infections, sarcoidosis, interstitial lung diseases, and even neoplastic lesions. Careful clinicopathological correlation is required to make an accurate diagnosis, particularly because pulmonary biopsies may yield non-specific findings [30].

Thyroid involvement in IgG4-RD, particularly in cases of Riedel thyroiditis or the fibrosing variant of Hashimoto’s thyroiditis, can clinically and radiologically mimic malignancy. Recognition of the characteristic fibrosing inflammation, in conjunction with increased IgG4-positive plasma cells revealed via immunohistochemical analysis, is essential to differentiate these conditions from neoplastic processes [30].

Hematologic manifestations of IgG4-RD, including generalized lymphadenopathy, peripheral eosinophilia, and polyclonal hypergammaglobulinemia, may resemble systemic hematologic conditions such as multicentric Castleman disease, lymphoma, and hypereosinophilic syndromes. As these entities often require substantially different management, tissue diagnosis supported by histology and immunohistochemistry is essential for accurate classification [5].

## 5. Discussion

Despite significant advances in characterizing the histopathological hallmarks of IgG4-RD, achieving definitive diagnosis remains a complex task. This complexity arises from the disease’s clinical heterogeneity, overlapping features with other fibroinflammatory and neoplastic conditions, and limitations in the specificity of current diagnostic tools. Moving forward, the diagnostic landscape will benefit from concerted efforts to refine and harmonize histological criteria, particularly by establishing organ-specific thresholds and enhancing laboratory standardization protocols [31].

Emerging histotechnological innovations hold substantial promise for improving tissue-based diagnostics. Techniques such as multiplex immunohistochemistry and digital pathology now enable high-resolution, quantitative assessments of immune cell populations and fibrosis architecture, offering greater diagnostic granularity. Additionally, in situ hybridization has shown value as a complementary approach, particularly in cases where conventional immunohistochemistry is inconclusive or compromised by sample quality [32]. These technologies could mitigate the limitations of standard tissue processing and staining, enhancing reproducibility across institutions.

Concurrently, advances in immunology and molecular biology are poised to transform diagnostic approaches toward less invasive and more specific methodologies. The identification of circulating plasmablasts, IgG4-specific autoantibodies, and distinct non-IgG4 immunoglobulin profiles may offer enhanced sensitivity for early detection. Notably, the discovery of CD4^+^ SLAMF7^+^ cytotoxic T cells as critical mediators of IgG4-RD pathogenesis introduces a novel immunophenotypic target for diagnostic assay development [33]. These findings suggest a broader immunopathological framework beyond the traditional IgG4-centric paradigm.

Furthermore, multi-omics technologies integrating genomics, transcriptomics, and proteomics offer an unprecedented opportunity to unravel the molecular complexity of IgG4-RD. Such approaches may enable disease subclassification, facilitate risk stratification, and support the identification of preclinical disease states [34]. The incorporation of these molecular insights into diagnostic workflows could redefine current classification models and guide personalized management strategies.

Artificial intelligence (AI) applications—particularly machine learning algorithms applied to histopathological imaging—could revolutionize diagnostic accuracy. By integrating clinical, serological, and pathological data, AI-driven platforms may assist in recognizing subtle diagnostic patterns and reduce interobserver variability [21]. This integrative, data-rich approach holds significant promise for achieving earlier and more precise diagnoses, especially in resource-limited or high-burden settings.

## 6. Conclusions

IgG4-RD presents a persistent diagnostic challenge due to its diverse clinical manifestations, multisystem involvement, and histopathological overlap with both malignant and non-malignant inflammatory conditions. Diagnosis relies heavily on the identification of hallmark histological features—namely, dense lymphoplasmacytic infiltrates, storiform fibrosis, and obliterative phlebitis—alongside an increased number of IgG4-positive plasma cells, as stated in the 2019 ACR/EULAR classification criteria. However, the limited sensitivity of serum IgG4 levels and variability in biopsy sampling highlight the indispensable role of histopathology and immunohistochemistry in establishing accurate diagnosis.

The complexity of IgG4-RD demands a multidisciplinary approach whereby pathologists, rheumatologists, and radiologists collaborate closely to interpret clinical, radiological, and tissue-based findings. While conventional diagnostic strategies provide a foundation, recent advances in molecular diagnostics, the identification of novel biomarkers such as circulating plasmablasts and disease-specific autoantibodies, and the integration of technologies like in situ hybridization and AI-driven digital pathology are paving the way toward more precise and standardized diagnostic pathways.

Despite the inherent diagnostic difficulties posed by this protean fibroinflammatory disorder, continuous research and innovation are gradually enhancing our understanding and capabilities. Strengthening interdisciplinary collaboration and incorporating emerging diagnostic tools will be essential to improve accuracy, reduce misdiagnosis, and ultimately optimize patient outcomes in IgG4-RD.

## Figures and Tables

**Table 1:** Pathologist’s Role in the 2019 ACR/EULAR Classification Criteria for IgG4-RD.

Diagnostic Phase	Pathologist’s Responsibility	Criteria Component		Points
Step 1: Entry criteria	Confirm histologic evidence	Inflammatory process with lymphoplasmacytic infiltrate in ≥1 organ (e.g., pancreas, salivary glands, bile ducts, orbits, kidney, lung, aorta, retroperitoneum, pachymeninges, or thyroid gland)		Mandatory for proceeding
Step 2: Exclusion criteria	Rule out mimics	Cellular infiltrates suggesting malignancy (e.g., lymphoma).		Disqualifies if present
		Inflammatory myofibroblastic tumor (ALK1+ markers).		
		Prominent neutrophilic inflammation or necrosis.		
		Necrotizing vasculitis.		
		Granulomatous inflammation.		
		Histiocytic/macrophage disorders.		
Step 3: Inclusion criteria	Histopathology Scoring	Uninformative biopsy		0
		Dense lymphoplasmacytic infiltrate (DLI).		4
		DLI + obliterative phlebitis.		6
		DLI + storiform fibrosis.		13
	Immunostaining Scoring	IgG4+:IgG+ ratio	IgG4+ cells/hpf	
		0–40% (or indeterminate)	0–9	0
		≥41%	0–9 (or indeterminate)	7
		0–40% (or indeterminate)	≥10 (or indeterminate)	
		41–70%	≥10	14
		≥71%	10–50	
		≥71%	≥51	16
Step 4: Final classification	Collaborate with clinicians to correlate findings	Combined histopathology, immunostaining, serum IgG4, and imaging scores. ≥20 points: definite IgG4-RD diagnosis.		

**Table 2:** Key Histopathologic Features of IgG4-Related Disease (IgG4-RD).

Histological Feature	Description	Histological Microphotography
Dense lymphoplasmacytic infiltrate (A)	Dense lymphoplasmacytic infiltrate with associated fibrosis, characteristic of IgG4-related disease.	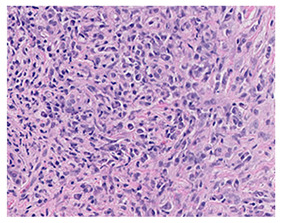
Storiform fibrosis (B)	Storiform (cartwheel-like) arrangement of spindle cells and collagen bundles with interspersed lymphoplasmacytic inflammation.	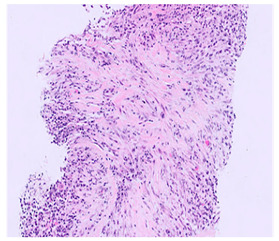
Obliterative phlebitis (C)	A vein is infiltrated and effaced by dense lymphoplasmacytic inflammation, leading to complete obliteration of the lumen (left). The EVG stain shows destruction of the vessel wall, confirming obliterative phlebitis (right). This finding is pathognomonic but not always present.	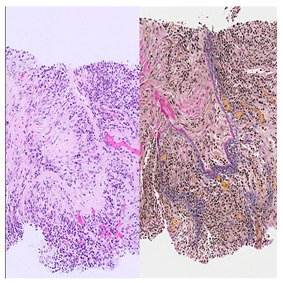
Increased IgG4-positive plasma cells (D)	Elevated IgG4+ plasma cells (>10 cells/HPF in most organs; higher thresholds for the pancreas and kidney) support the diagnosis but are not sufficient alone. In this case, IgG4-positive plasma cells account for >40% of the total IgG-positive plasma cell population, which represents a major diagnostic criterion in the 2019 ACR/EULAR classification for IgG4-related disease.	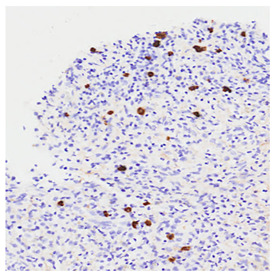

A. Dense lymphoplasmacytic infiltrate (H&E, ×400). B. Storiform fibrosis with interspersed lymphoplasmacytic inflammation (H&E, ×100). C. Obliterative phlebitis. H&E stain (left) shows a vein with dense lymphoplasmacytic infiltrate effacing the vessel wall and obliterating the lumen (×100). Elastic van Gieson (EVG) stain (right) shows destruction of the vessel wall, confirming obliterative phlebitis (×100). D. Immunohistochemical stain for IgG4 highlights numerous IgG4-positive plasma cells (IgG4 IHC, ×400).
